# COMPARING PHYSICAL HEALTH CHALLENGES OF RURAL AND URBAN CAREGIVERS OF PEOPLE WITH DEMENTIA IN THE UNITED STATES

**DOI:** 10.1093/geroni/igae098.0060

**Published:** 2024-12-31

**Authors:** Fei Wang, E-Shien Chang, Phoebe Tran

**Affiliations:** University of Tennessee Knoxville, Knoxville, Tennessee, United States; Weill Cornell Medical College, New York, New York, United States; University of Tennessee Knoxville, Knoxville, Tennessee, United States

## Abstract

Increased dependence on informal caregiving and limited access to healthcare services in rural U.S. communities may contribute to worse physical health in rural informal caregivers of persons with dementia (PWD) relative to urban counterparts. We used nationally representative 2020-2022 Behavioral Risk Factor Surveillance Systems surveys to examine the association between rural/urban residence and poor physical health days in caregivers of PWD (N=17405). Self-reported physical health within previous month was categorized as follows: 0 poor physical health days (PPHD), 1-13 PPHD, 14+ PPHD. Information on rural/urban county of residence, sociodemographic characteristics, and caregiving factors were obtained from the surveys. Our results showed that 1 in 4 rural caregivers (25%) reported 14+ PPHD in the previous month as compared to 1 in 10 urban caregivers (10%). Findings from multinomial logistic models indicated that rural caregivers had 2.16 times the odds (OR: 2.16, 95% CI:1.03-4.53) of having 14+ vs 0 PPHD compared to urban caregivers after adjusting for sociodemographic characteristics and caregiving factors. These findings suggested prolonged physical health challenges for rural caregivers. In contrast, the odds of having 1-13 vs 0 PPHD were not significantly different between rural and urban caregivers (OR: 0.92, 95% CI: 0.53-1.62). Efforts to enhance long-term physical health for rural caregivers are warranted. Given that geographical isolation and limited resources in rural settings may impede physical health, community-based activities promoting physical activity and social connections should be prioritized. Policy advocacy is crucial to raise awareness of urban-rural health disparities and support targeted interventions for rural caregivers of PWD.

